# News

**DOI:** 10.1155/S1463924679000481

**Published:** 1979


### Liquid chromatography

An informative guide to high performance liquid chromatography columns and packings has recently been published by Du Pont. The brochure offers an opportunity to apply their technology to HPLC needs. It covers detailed performance and quality criteria, a sample column performance report and a helpful guide for proper column selection. It also features reverse phase column data, high performance size exclusion column specifications and a complete list of column parts and accessories which are available from Du Pont.

*Du Pont (UK) Ltd., 64a Wilbury Way, Hitchin, Herts.*

### Precision of test methods

The first part of a new British Standard which will be of fundamental importance in improving standardization practice has been published by the British Standards Institution. BS 5497 Precision of test methods is a new basic reference standard to be used as an aid to the quality control of bulk commodities and is issued complete with a computer programme for ease of application.

BSI has published over 1000 standard test methods, many of which include comprehensive test requirements, particularly where bulk commodities, agricultural products, chemical/fuels or raw materials are concerned. However, the establishment of standard methods alone does not guarantee that test results subsequently obtained can be accepted without reservation. Many different factors (apart from sampling error) may contribute to variability, eg, the operator, the instruments and the equipment used, the calibration of the equipment and environment (temperature, humidity, air pollution).

BS5497 Part 1 Determination of repeatability and reproducibility for standard test method describes the organisation and analysis of interlaboratory experiments for the numerical determination of two extreme measures of variability, repeatability and reproducibility. Repeatability refers to tests performed at short intervals in one laboratory by one operator using the same equipment, while reproducibility refers to tests performed in different laboratories, which implies different operators, different equipment and different times. This British standard has been published in an attempt to rationalize alternative practices published by various industrial sectors, since the accumulation of test data is often less crucial than the requirements to establish the integrity of the statistical analysis and agree upon the significance of any variability in the test results obtained. Consequently a precise step-by-step statistical procedure has been included in the standard along with a flow diagram and a computer programme, in FORTRAN, to assist implementation. It will be used in future instead of product committees establishing precision criteria for their own sectors.

*Copies of BS5497 Part 1, price £8.80, may be obtained from BSI Sales Department, 101 Pentonville Road, London N1 9ND.*

### News

### Summer school on automatic methods of analysis

Automated methods of analysis are becoming widespread and more capable of dealing with complex analytical problems. They range from the sophisticated and expensive to the simple and cheap. Consequently the laboratory manager has to be fully atuned to new developments in technique and the economic rationale of any method adopted.

The Summer school is concerned with these two aspects of automatic analysis and will pursue them in lectures, small group discussions and practical sessions.

The speakers include Professor R. Bartells, B. Denton, E. Hanson, H. Pardue and J. Ruzicka from abroad and Drs. D. Betteridge, D.R. Deans, J.K. Foreman, F.L. Mitchell, D. Porter, P.G. Sanders and P.B. Stockwell from the U.K. The new techniques they will cover are the automation of chromatography, kinetic spectroscopic and single test methods, flow injection analysis and the applications of microprocessors. In addition there will be several lectures on problems facing a laboratory manager who is contemplating introducing automation. A course problem has been devised to bring out these issues. It is the consideration of an important problem, which has been faced by the Laboratory of the Government Chemist viz the automated analysis of tobacco smoke.

The course organisers are Dr. D. Betteridge (University College, Swansea) Mr. D. Porter and Dr. P.B. Stockwell (Laboratory of the Government Chemist). The school is being organised under the auspices of the Chemical Society and further details are available from Dr. M.D. Robinson, The Chemical Society, Burlington House, Piccadilly, London W1V OBN.

### Automatic analysis

Traditionally the fields of food and nutrition have not emphasized automated analyses, but recent developments in the United States food labelling laws have brought about an increased interest in this area. The increased number of papers at the US national meetings of the Institute of Food Technologists, American Chemical Society, and Association of Official Analytical Chemists (AOAC) given on the automated and semiautomated analysis of nutrients in foods are indications of this increased interest. For example, the 92nd Annual Meeting of the AOAC featured an evening seminar devoted solely to the automated analyses of nutrients in foods. This seminar was organized by A. Conetta of Technicon Inc. and featured P.A. Lachance of Rutgers University, J.E. Vanderveen of the Food and Drug Administration, J.R. Kirk of the University of Florida and K.K. Stewart from the US Department of Agriculture. In addition the AOAC's new Committee on Automation has been active and hopes to increase the acceptance of automation in the regulatory laboratories in the US Development of automatic methods of analysis of nutrients in foods presents some special problems to the analytical chemists. Food stuffs are complex, usually multiphasic, and often contain thousands of chemical compounds. Few of the existing laboratories use automated methods, and equipment budgets are limited. At the same time there are needs for automated methods. There are about 4000 food items in the US and 60,000 individual foods, counting brand names. Analyses are needed in many areas, eg, quality control, nutrient data banks and regulatory actions.

Interested analytical chemists might consider working in this area. The US Drug Administration, Food and Drug Administration and National Science Foundation have some limited extramural research and contract money for studies in this area.

### Consultancy Service

SIM Datasystems have many years experience in the use of small computers for medical and industrial laboratories; covering systems analysis, programming, interfacing and installation. The company offers a full consultancy service extending from systems design through to implementation of the computer and staff training. Complete computer systems including hardware, programs and interfacing are available from SIM Datasystems for a wide range of applications in chemical pathology.

***Further information can be obtained from SIM Datasystems, Fishponds Road, Wokingham, Berks RG11 2QA, UK.***

